# Reply to: Amplifying Women's Voices in Menopause Research: The Importance of Inclusive Perspectives [Letter]

**DOI:** 10.1111/hex.70189

**Published:** 2025-02-18

**Authors:** Camille Cronin, Sara Donevant, Kerri‐ann Hughes, Marja Kaunonen, Jette Marcussen, Rhonda Wilson

**Affiliations:** ^1^ School of Health and Social Care University of Essex Colchester Essex UK; ^2^ College of Nursing University of South Carolina Columbia South Carolina USA; ^3^ School of Nursing Massey University Palmerston North New Zealand; ^4^ School of Health Sciences Tampere University Tampere Finland; ^5^ Department of Applied Health Sciences UCL University College Odense Denmark; ^6^ Department of Nursing RMIT University Melbourne Australia

## Author Contributions

**Camille Cronin:** conceptualization, writing–original draft, writing–review and editing, project administration. **Sara Donevant:** writing–original draft. **Kerri‐ann Hughes:** writing–original draft. **Marja Kaunonen:** writing–original draft. **Jette Marcussen:** writing–original draft. **Rhonda Wilson:** conceptualization, writing–review and editing, supervision.

## Ethics Statement

The authors have nothing to report.

## Consent

The authors have nothing to report.

## Conflicts of Interest

The authors declare no conflicts of interest.

Dear Editor,

Thank you for your thoughtful engagement with our article “Amplifying Women's Voices in Menopause Research: The Importance of Inclusive Perspectives.” The response to this publication on the critical importance of incorporating women's voices in menopause research is very much appreciated.

Your insightful points are acknowledged and welcome the opportunity to clarify this approach. This article was intended as a viewpoint piece, designed to stimulate discussion and encourage researchers to further explore this area within their own work. While extensive research has been conducted in related fields, the aim was to highlight gaps and inspire broader consideration of inclusion in menopause research.

Regarding your specific suggestions:

1. Case studies and practical examples: We agree that detailed case studies, indeed case study research design [1] could provide valuable illustrations and mixed method approaches aligned with inclusive research in practice. While this viewpoint piece did not focus on specific case studies, future research could certainly expand on this aspect to offer concrete examples of how inclusivity improves outcomes.
2. Challenges and strategies for inclusion: This is an important point raised about the barriers to incorporating women's voices. While the discussion acknowledged the need for diverse research methods, further exploration of the obstacles and potential solutions would undoubtedly strengthen the discourse. This perspective is appreciated and certainly would encourage future studies to delve deeper into these challenges.
3. Quantitative data and methodological details: While this article primarily utilized qualitative insights as referenced to emphasize experiential perspectives, the value of quantitative data in complementing these findings is fully recognized. This is why the article encourages the need to include “the voice of women” in co‐designing further research efforts to integrate both qualitative and quantitative approaches that enhance the depth and reliability of studies in this field.

Once again, your engagement and constructive feedback is much appreciated. It is hoped that this article serves its purpose in sparking meaningful discussions and inspiring further inquiry into inclusive menopause research. Thank you for contributing to this important dialogue.

## Patient or Public Contribution

The content of the viewpoint article was informed by a menopause service user group who discuss their experiences of menopause and meet on a regularly to co‐design and co‐produce activities that inform our ongoing research on menopause.

## Data Availability

The authors have nothing to report.
